# Assessment of a treatment guideline to improve home management of malaria in children in rural south-west Nigeria

**DOI:** 10.1186/1475-2875-7-24

**Published:** 2008-01-29

**Authors:** Ikeoluwapo O Ajayi, Catherine O Falade, E Afolabi Bamgboye, Ayo MJ Oduola, Oladele O Kale

**Affiliations:** 1 Department of Epidemiology, Medical Statistics and Environmental Health, College of Medicine, University of Ibadan, Oyo State, Nigeria; 2 Department of Pharmacology and Therapeutics, College of Medicine, University of Ibadan, Oyo State, Nigeria; 3 Special Programme for Research and Training in Tropical Diseases, World Health Organisation, Geneva, Switzerland

## Abstract

**Background:**

Many Nigerian children with malaria are treated at home. Treatments are mostly incorrect, due to caregivers' poor knowledge of appropriate and correct dose of drugs. A comparative study was carried out in two rural health districts in southwest Nigeria to determine the effectiveness of a guideline targeted at caregivers, in the treatment of febrile children using chloroquine.

**Methods:**

Baseline and post intervention knowledge, attitude and practice household surveys were conducted. The intervention strategy consisted of training a core group of mothers ("mother trainers") in selected communities on the correct treatment of malaria and distributing a newly developed treatment guideline to each household. "Mother trainers" disseminated the educational messages about malaria and the use of the guideline to their communities.

**Results:**

Knowledge of cause, prevention and treatment of malaria increased with the one-year intervention. Many, (70.4%) of the respondents stated that they used the guideline each time a child was treated for malaria. There was a significant increase in the correct use of chloroquine from 2.6% at baseline to 52.3% after intervention among those who treated children at home in the intervention arm compared with 4.2% to 12.7% in the control arm. The correctness of use was significantly associated with use of the guideline. The timeliness of commencing treatment was significantly earlier in those who treated febrile children at home using chloroquine than those who took their children to the chemist or health facility (p < 0.005). Mothers considered the guideline to be explicit and useful. Mother trainers were also considered to be effective and acceptable.

**Conclusion:**

The use of the guideline with adequate training significantly improved correctness of malaria treatment with chloroquine at home. Adoption of this mode of intervention is recommended to improve compliance with drug use at home. The applicability for deploying artemisinin-based combination therapy at the community level needs to be investigated.

## Background

In Nigeria, the main strategy for reducing childhood morbidity and mortality is presumptive treatment of all fevers in children under five with antimalarial drugs [1]. This is in line with World Health Organisation (WHO) recommendation for endemic countries where the availability and use of laboratories are limited [2].

Malaria control activities in Nigeria are planned and implemented through the Primary Health Care (PHC) system [1]. However, use of health centres, as the first resort for malaria management has been shown to be low in many African studies including Nigeria. It is estimated that only about 20% of malaria episodes are treated in the health centres [3]. A review of mothers' malaria treatment-seeking behaviour in rural south-western Nigeria revealed that more than 80% of malaria episodes received treatment outside of the existing government healthcare system [4,5]. Reasons underlying this practice include difficulty with access to health centre, scarcity of affordable drugs including antimalarial drugs, perceived deficiencies in the performance of formal health services including poor clinical skills, attitude of health personnel and cultural beliefs [6,7]. These shortcomings encourage treatment of malaria at home with drugs bought from shops and herbal preparations [8,9]. These treatments are usually incorrect or sub-optimal [10-12]. Mothers and caregivers only usually visit a health centre or hospital after the illness has failed to respond to several drugs and ineffective self treatment. This practice increases morbidity and mortality in addition to contributing to possible emergence of drug resistance [6,13].

Prompt and effective treatment of malaria remains a challenge for malaria control programmes. From the moment a patient experiences the first symptom of malaria to the point when the disease has been cured; many potential barriers have to be overcome. These include delays in recognition of symptoms and treatment seeking, and non-adherence to the drug regimen. Helping caregivers to overcome these and other barriers to prompt and effective treatment of malaria is of particular importance in sub-Saharan Africa.

Despite the considerable success recorded from past interventions to improve compliance to correct doses of chloroquine (CQ) in uncomplicated malaria [9,13-17], the practice of inappropriate and incorrect home management of malaria still persists in many communities especially in the rural areas. Another observation is that most of the interventions were pre-determined by the investigators with little or no input from the members of the communities. Furthermore, investigators often supplied the antimalarial drugs used (usually chloroquine), with no adequate mechanism put in place to ensure that the same quality of drugs used for the studies continued to be available in the community.

Efforts during this study was devoted to determining the baseline knowledge and practice of mothers/caregivers in the management of malaria in children, to providing training on correct dosage and regimen of the drug of choice for treating malaria (chloroquine) at the time of the study and evaluating the effectiveness of a newly designed treatment guideline.

## Methods

### Study design

A comparative study was carried out in two rural health districts ; one health district belonged to the intervention arm while the other was the control arm. Outcome measures were evaluated in both arms before and after intervention. The study was conducted in three phases. The first phase involved advocacy, community mobilization, baseline survey and selection of "mother trainers". Information, Education and Communication (IEC) materials were developed and research staff recruited and trained. The intervention phase involved the development of a treatment guideline, training of "mother trainers" and distribution of the guideline. A year after commencement of guideline distribution, a household survey, interviewing eligible caregivers about management of childhood fevers in the preceding two weeks, was performed to evaluate the intervention.

A longitudinal follow-up study was nested into this study in order to collect data on treatment outcome (reported in a separate manuscript). The nested study entailed daily visits of study team to identify children with febrile illness, ask questions about treatment practices and collect finger pricked blood sample for microscopy on Days 0,1,2,3,7 and 14. These visits were carried out for nine months. The main study continued for another three months prior to post intervention survey.

### Ethics

The Joint University of Ibadan/University College Hospital Ethical Committee provided ethical approval for the study. Permission to carry out the study in the selected villages was obtained from the village heads and community opinion leaders. Heads of households or their representatives gave permission for household survey. Individual verbal informed consent was also obtained from each caregiver interviewed.

### Study location

This study was conducted in two rural districts selected by random sampling from the eight health districts that make up Ona-Ara Local Government area (LGA), in south western Nigeria from March 2002 to October 2004. The two health districts were allocated into intervention or control arm by balloting. Ona-Ara LGA is one of 33 LGAs in Oyo State. It is located about 35 km east of Ibadan, the capital of the state and has 26,976 households with a population of about 147,847 people, out of which 33,126 are children and 33,576 are women of childbearing age (Projected figure, 1997). Ona-Ara LGA comprises 200 villages that are divided into 8 health districts [18], six of which are rural and two, are urban/peri-urban. Each of the two health districts has a health/maternity centre and a primary health centre is located at Akanran the headquarters of the LGA.

The inhabitants are mainly peasant farmers and traders from the Yoruba ethnic group. The climate is that of tropical rainforest zone, with a warm dry-season from November to April and a rainy season from May to October. Malaria is hyper-endemic in the LGA with perennial transmission. Home-treatment of malaria using drugs purchased from private outlets is common.

### Study population

Mothers or female caregivers who have resided in the community for at least one year, have at least a child ≤ 10 years and consented to participate in the study were enrolled. Mothers or female caregivers who provided care to children only on short term basis such as day care settings and children with severe illness were excluded.

#### Sample size

The sample size for the survey was calculated using compliance rate of 25% for chloroquine when used at home to treat febrile illness presumed to be malaria [19]. To have a 90% power of detecting a significant effect if the intervention increases the proportion of correct use of CQ by 50%, assuming a design effect of two, the minimum number of children required in each group was 280. These were selected from the study districts using probability proportional to size sampling technique.

### Intervention

Mothers numbering two to 10 per village depending on size of the village (one per 50 households) were selected by the community to be trained as "mother trainers". Selected "mother trainers" were trained to form a core group of mothers to provide education on malaria diagnosis, treatment and prevention to other caregivers/mothers in their communities. During the training, "mother trainers" understanding of the local terminology *'Iba' *and the researchers' terminology for malaria was clarified. It was made clear that malaria in the Yoruba language is consistent with *"iba" *and is recognized by symptoms associated with malaria such as fever, vomiting, chills, anorexia, weakness, yellow urine and headache as reported by Falade et al. [17].

**Figure 1:**
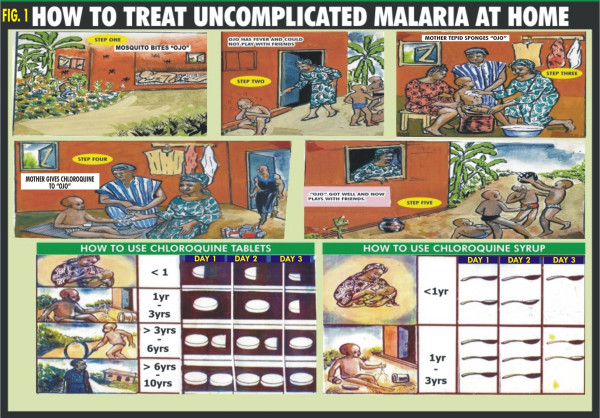
**How to treat uncomplicated malaria at home**. -Scenario showing the presentation and treatment of uncomplicated malaria in the home.

**Figure 2:**
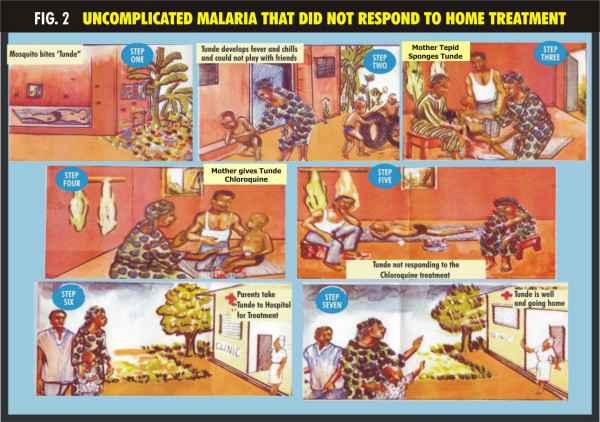
**Uncomplicated malaria that did not respond to home treatment**. -Scenario showing the presentation and management of uncomplicated malaria that did not respond to home treatment.

**Figure 3:**
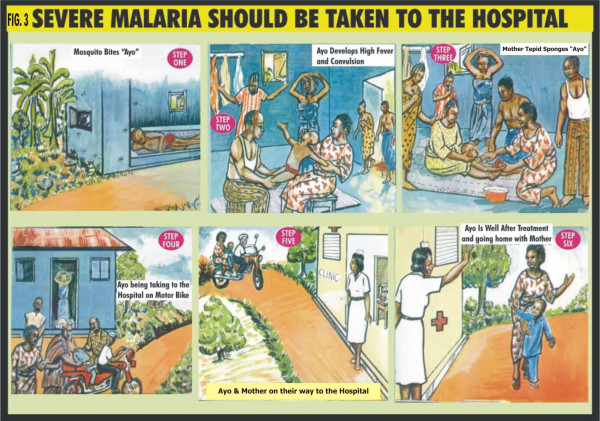
**Severe malaria should be taken to the hospital**. -Scenario showing the presentation and management of severe malaria.

A treatment guideline was developed using participatory approach involving "mother trainers" and community members. Pictorial illustrations of some common clinical features of uncomplicated and complicated malaria, the steps to take for the different types of presentation of malaria and the correct dose and regimen of chloroquine treatment according to the age of the child were used and presented as cartoons (Figures 1, 2 &3). Chloroquine was the first drug of choice for malaria treatment in Nigeria at the time of this study. The Federal Ministry of Health, Nigeria adopted artemisinin-based combination therapy (ACT) as first line drug for uncomplicated malaria in line with the WHO recommendation in January 2005 [1].

The developed treatment guideline was distributed by mother trainers to every household in their communities and some were pasted on the walls of prominent buildings in the communities. Information on how to use the guideline was also provided. Drug sellers in the intervention arm of the study were also trained about the correct management of malaria using CQ and the guideline was distributed to them too.

No form of intervention was carried out in the communities in the control arm of the study. Mothers were left to continue their usual treatment practices for malaria. They were not provided with the treatment guideline. With the collaboration of the Primary Health Care unit of the LGA, the site was monitored to ensure that no form of project related to malaria or that, which may confound findings in this study was carried out. It was planned that if the intervention was found effective, it will be introduced into the communities in the control arm of the study.

### Evaluation

Twelve months after commencement of distribution of the guideline, a two week fever recall household survey among study population was carried out to evaluate the intervention. The primary outcome which was correctness of use of chloroquine was measured using caregivers' recall of dose of chloroquine given to febrile children as well interval and duration for which it was given.

### Data collection methods

An investigator administered questionnaire was used to collect data for the household survey. The household survey focused on the caregivers' knowledge about malaria and their health seeking behaviour for fever in children presumed to be malaria.

The questionnaire used was adapted from a related past study conducted in Togo [20] and complementary questions were added using findings from pilot focus group discussions. The questionnaire was translated from French to English and back translated to French by independent translator to ensure correct translation [see Additional file 1], The questionnaire was then translated into "Yoruba" (local language in the study area) and back translated to English by an independent translator. The questionnaire was face validated and tested for reliability using test-retest reliability method. The reliability co-efficient was 0.85 at 5% level of significance.

### Drug supply

Mothers were requested to buy chloroquine from their usual drug sources. Most mothers in this study area usually purchase chloroquine from drug hawkers and patent medicine sellers (PMS).

### Data analysis

Data entry and statistical analysis were conducted using EPI info version 6.02. Chi Square test for categorical variables, t-test for continuous variables, and non-parametric test (Kruskal-Wallis test) were used to investigate associations, compare proportions and compare means respectively at 5% level of significance.

## Results

Six hundred and eleven (281 in control and 330 in the intervention group) mothers/caregivers were studied at baseline. The mean ages (SD) of the mothers were 37.1 (13.3) and 37.5 (13.9) years respectively, (p = 0.73). Six hundred and four mothers (304 in control and 300 in the intervention groups), with mean (SD) ages 34.5 (13.3) and 36.6 (3.4) years respectively, p = 0.09 were interviewed during post-intervention survey.

### Impact of intervention on mothers' knowledge of symptoms and signs, cause, prevention and treatment of malaria

At baseline, 13 of the 330 (3.9%) mothers knew the correct cause of malaria while 228 of 300 (76.0%) mentioned mosquito bites after the intervention (Table 1). Chemoprophylaxis was the most common method of prevention of *"Iba" *mentioned by 143/171 (80.9 %) and 185/255 (72.5%) pre- and post- intervention respectively. Further details are shown in Table 1. The drug mostly mentioned as being the most important in treating malaria were antimalarial drugs comprising chloroquine and sulphadoxine-pyrimethamine commonly Fansidar^® ^mentioned by 162/324 (50.0%) and 217/296 (73.3%) pre and post intervention respectively, (χ^2 ^= 68.91, df = 5, p = 0.000). Further details are shown in Table 1. None of the respondents mentioned artemisinin-based antimalarial drug. There was no significant difference in responses to these variables pre and post intervention in the control group (p > 0.05).

**Table 1:** Table showing improvement in mothers' knowledge on cause of malaria, prevention and drug most important in the treatment of malaria.

	Intervention arm only		
	Baseline (n = 330)	Post intervention (n = 300)	Level of significance

*Perceived cause of malaria			

Playing in the sun	144 (43.6%)	87 (29.0)	X^2 ^= 13.87; p = 0.0002
Playing in the rain	48 (14.5%)	32 (10.7%)	X^2 ^= 1.80; p = 0.18
Drinking dirty water	21 (6.4%)	18 (6.0%)	X^2 ^= 0.0; p = 0.98
Mosquito bite	13 (3.9%)	228 (76.0%)	X^2 ^= 342.4; p < 0.0001
Inherited	15 (4.5%)	9 (3.0%)	X^2 ^= 0.65; p = 0.42
Too much work	9 (2.7%)	7 (2.3%)	X^2 ^= 0.0; p = 0.95
Dirty surroundings	24 (7.3%)	18 (6.0%)	X^2 ^= 0.23; p = 0.63
Eating too much palm oil	8 (2.4%)	3 (1.0%)	X^2 ^= 1.12; p = 0.29

Knowledge of prevention			

Total number that said malaria can be prevented	177 (53.6%)	255(85%)	X2 = 41.62; p < 0.0001

*Prevention methods known			

Chemoprophylaxis	143 (80.8%)	185 (72.5%)	X^2 ^= 0.25; p = 0.62
Prevent water puddles	8 (4.5%)	35 (13.7)	X^2 ^= 8.88; p = 0.002
Bednets	5 (3.0%)	31 (12.2%)	X^2 ^= 10.72; p = 0.001
Insecticides	10 (5.6%)	3 (1.2%)	X^2 ^= 5.71; p = 0.02
Barrier cream	5 (3.0%)	3 (1.2%)	X^2 ^= 0.79; p = 0.28
Avoid sun, rain and dust	3 (1.7%)	2 (0.7%)	X^2 ^= 0.0; p = 1.0
Drug most important for treatment of malaria	N = 324	N = 296	
Antimalarial drugs (CQ, Fansidar^®^)	162 (50.0)	217 (73.3%)	X2 = 68.91; p < 0.0001
Paracetamol	117 (36.1)	73 (24.7%)	
Haematinics/vitamins	16 (4.9)	4 (1.4%)	
NSAIDs	1 (0.3)	0 (0.0%)	
Antibiotics	5 (1.5)	2 (0.7%)	
Don't know	23 (7.1)	0(0.0%)	

* Multiple responses

### Effect of intervention on the care seeking practices of mothers for febrile illness presumed to be malaria

A total of 513 children (247; intervention and 266; control group) had an episode of fever in the three months preceding post intervention survey. In some households more than one child under 10 years had fever during this period. Further documentation on the management of febrile illness was carried out for 459 (89.5%) children (229; intervention and 230; control group) who had fever two weeks preceding the survey. The frequency distribution of the presumed diagnosis of febrile illness is shown in Table 2. A significant change was demonstrated after intervention (χ^2 ^= 17.5, df = 5, p = 0.004), with more mothers in the intervention arm presuming febrile illness to be malaria ("iba") than those in the control arm. However, the proportion of mothers who presumed "iba" as diagnosis in the intervention arm was the same at pre- and post- interventions. The types of first treatment given to a febrile child in the two arms are shown in Table 3. Treatment at home with orthodox drug was the most mentioned in both arms. Details of observed changes in both study arms pre and post intervention are shown in Table 3.

**Table 2:** Frequency distribution of presumed diagnosis of febrile illness in the child

**Diagnosis**	**BaselineIntervention**		**Post intervention**	
	
	**Intervention ** **N = 322 n (%)**	**Control ** **N = 257 n (%)**	**Intervention ** **N = 229 n (%)**	**Control** ** N = 230 n (%)**
"Iba" (Malaria)	267(82.9)	222(86.4)	189(82.5)	173(75.2)
"Iba Ponju" (Yellow fever)	1(0.3)	1(0.3)	6(2.6)	21(9.1)
"Iko ile tutu" (Pneumonia)	4(1.2)	0(0.0)	10(4.4)	16(7.0)
"Ogbele" (Measles)	6(1.9)	2 (0.8)	2(0.9)	6(2.6)
"Cough"	2(0.6)	3(1.2)	-	-
Don't know	20(6.2)	20(7.8)	-	-
Others	22(6.8)	9(3.5)	22(9.6)	14 (6.1))

	**X2 = 5.07; p = 0.28**		**X2 = 17.5; p = 0.004**	

**Table 3:** The distribution of first treatment given to the child at home during last episode of febrile illness.

	**Baseline**		**Post intervention**	
	
**First treatment**	**Intervention** ** n (%)**	**Control** ** n (%)**	**Intervention** ** n (%)**	**Control** ** n (%)**
Gave drugs bought from chemist at home	233 (71.5)	185 (73.1)	183 (79.9)	141 (61.3)
Gave traditional herbs at home	37 (11.3)	29 (11.5)	10 (4.4)	32 (13.9)
Took child to health facility	37 (11.3)	23 (9.1)	21(9.2)	41(17.8)
Took child to chemist/PMS	18 (5.5)	13 (5.1)	15 (6.6)	16 (7.0)
Took child to traditional healer	1 (0.3)	3 (1.2)	0 (0.0)	0 (0.0)

**Total**	**326 (100)**	**253 (100)**	**229(100)**	**230(100)**

	**X2 = 2.39; p = 0.67**		**X2 = 23.45; p < 0.0001**	

The types of drugs bought by mothers who gave drugs at home are shown in Table 4. Post – intervention, the most common drug bought for treating malaria in the intervention arm changed from paracetamol (PCM)/PCM derivatives to chloroquine (p = 0.003) while it still remained as PCM/PCM derivatives in the control arm (p = 0.17).

**Table 4:** Frequency distribution of drugs bought from the chemist/itinerant drug sellers

**Name of drug**	**Intervention**		**Control**	
	
	**Baseline** **N = 189** **n (%)**	**Post** **N = 178** **n (%)**	**Baseline** **N = 141** **n (%)**	**Post** **N = 134** **n (%)**
Paracetamol	*140(74.1)	106 (59.5)	*103 (73.0)	*104 (77.6)
Vitamins/blood tonic	109 (57.7)	128 (71.9)	65 (46.1)	84 (62.7)
Chloroquine	85 (45.0)	*130 (73.0)	43 (30.5)	49 (36.6)
Antibiotics	21 (11.1)	18 (10.1)	10 (7.1)	17 (12.7)
"Akapo"^1^	18 (9.5)	9 (5.1)	18 (12.8)	12 (8.9)
Chlorpheniramine	6 (3.2)	10 (5.6)	6 (4.3)	2 (1.5)
Aspirin	5 (2.6)	3 (1.7	6 (4.3)	4 (3.0)

	**X^2 ^= 19.88; p = 0.003**		**X^2 ^= 9.15; p = 0.17**	

Note: There were multiple responses. * = The most mentioned in each group

In the intervention arm, one hundred and sixty-seven of 202 (82.7%) caregivers who treated febrile children at home bought drugs from hawkers at baseline. This was followed by PMS/chemist mentioned by 30 (14.4%) and health centre by 5(2.5%) caregivers (χ^2 ^= 32.11, df = 2, p = 0.0001). After intervention, 104/179 (58.1%) mothers in the intervention arm mentioned they bought drugs from PMS/chemist, 70 (39.1%) from drug hawkers and 5 (2.8%) from health centres/dispensary. There was no significant difference in the source of drug post intervention in the control arm (p = 0.59).

### Awareness and use of guideline

The findings on the awareness and perceived usefulness of the guideline by mothers irrespective of the treatment option used are summarized in Table 5. None of those in the control group mentioned they used a guideline. In the intervention group, 125 out of 183 (68.3%) of those who treated their children at home using orthodox drugs irrespective of drug used mentioned that they used the guideline in treating the sick children. Three out of 15 (20.0%) and 6/21 (28.6%) mothers who took their children to a chemist or health facility respectively mentioned that they used the guideline.

**Table 5:** Summary of mothers' responses to variables on awareness and usefulness of the guideline. (Intervention group)

**Variables**	**Responses**	**Frequency (%)**
Did you see the guideline distributed to mothers on treatment of malaria? (**N = 300**)	Saw the guideline	264 (88.0%)
Do you have the treatment guideline in your household? **(N = 300)**	Had the calendar type in their household	204 (68.0%)
Did you use the guideline? (**N = 204**)	Used guideline each time a child had "iba"	145(71.1%)
	Did not use guideline all the time child had "iba"	59 (28.9)
Why did you not use the guideline? (**N = 59**)		
	- Did not treat child with chloroquine	14 (23.7%)
	- had training on treatment before	10 (16.9%)
	- Child reacts to chloroquine	10 (16.9%)
	- Forgot to use guideline	7 (12.0%)
	- No response	18 (30.5%)
Was the guideline useful? (**N = 204**)		
	Very useful	168 (82.4%)
	Useful	23 (11.3%)
	Undecided	11 (5.4%)
	Not useful	2 (0.9%)
What benefits did you derive from using the guideline? (**N = 204**)		
	Provided knowledge on correct dose of chloroquine	188 (92.2%)
	Taught mothers to keep environment clean	8 (3.9%)
	- Guideline was free	8 (3.9%)
What difficulties did you experience in using the guideline? (**N = 14**)		
	Could not comprehend it	11 (78.6%)
	Kept forgetting the instructions	3 (21.4%)

### Correctness of use of chloroquine

At baseline, 3/116 (2.6%) (intervention) and 3/72 (4.2%) (control) caregivers who used chloroquine at home used it correctly. None of those who took their children to chemist or health facility in the control and intervention arm and was prescribed CQ used it correctly.

After intervention, chloroquine was used correctly in terms of dose and duration among caregivers who treated children at home with drugs in 69/132 (52.3%) and 9/57 (15.8%) cases in intervention and control arm respectively, χ^2 ^= 20.38; p < 0.001. When compared with baseline findings [3/116 (2.6%) versus 69/132(52.3%)] there was a significant positive change in the correct use of chloroquine in the intervention group [OR = 0.02, 95% CI 0.00–0.008], (p < 0.001) while there was no significant change in the control group [3/72 (4.1%) versus 9/57(15.8%)], (p = 0.05).

Post intervention, 3/15 (20.0%) respondents in the intervention group who took their children to a chemist/PMS and used CQ, used it correctly while none of the mothers in the same category in the control group used it correctly. Two out of 26 (7.7%) who took their sick children to health facility used it correctly. This comprised of one each in intervention [1/9 (11.1%)] and control group [1/17 (5.9%)].

Use of the guideline was significantly associated with correctness of use of CQ, (p < 0.001) [OR = 44.63, 95% CI 7.63–433.4]. In the intervention arm, 42 out of 50 respondents (84.0%) who used chloroquine following the guideline administered chloroquine correctly, compared to two out of 19 (10.9%) who did not use the guideline (p < 0.001). Also, use of the guidelines was significantly associated with the level of education (χ^2 ^= 4.91, p = 0.03). Caregivers who had at least primary education constituted the largest percentage of those who used the guidelines [85/145;58.6%] while those who did not have any education were the most among those who did not use the guidelines [45/59;76.3%]. There was no significant association between use of the guidelines and other demographic characteristics

### Timeliness of treatment

All the 230 caregivers in the intervention group and 182 in the control group who treated with orthodox drugs at home at baseline commenced treatment within 24 hours of mother noticing fever while 176/178 (98.9%) and 138/139 (99.3%) respectively did so post intervention. The proportion of mothers who took sick children to the chemist within 24 hours of noticing fever increased from 17/18 (94.4%) at baseline to 15/15 (100%) post intervention in the intervention arm (p = 0.93) while 6/7 (85.7%) and 10/12 (83%) did so at baseline and post intervention respectively in the control arm (p = 0.61). There was a reduction in the proportion of mothers who took febrile children to a health facility within 24 hours of noticing fever from 20/26 (76.9%) to 15/21 (71.4%) in the intervention arm (p = 0.67) and 17 (100%) to 30/41 (73.2%) in the control arm (p = 0.02).

### Microscopic diagnosis and treatment outcome

Thick blood smears results were available on D0 for 162 febrile children [88, control arm and 74 intervention]; 64/88 (72.7%) and 62/74 (83.7%) had patent parasitaemia in the control and intervention arms respectively. Parasite density ranged from 40/μl to 120,000/μl with only nine children with a parasite density < 800 parasites/μl. The sensitivity and specificity of caregivers' diagnosis were 78.1% and 29.2% versus 82.3% and 8.3% in the intervention and control arms respectively. Complete blood smear reports were available for 49 and 46 children in the control and intervention arm respectively. Day 14 cure rates were 63.3% (31/49) and 91.3% (42/46) in the control and intervention arms (p < 0.001).

### Referrals to the health facilities during the one year intervention period

Six of 183 febrile children treated at home in the intervention arm and 15/141 (10.6%) in the control arm were taken by mothers/caregivers to health care facility (HCF)/chemist, (p = 0.009). Children were taken to HCF/chemist because of failure to improve mentioned by 17 (81.0%) respondents and need to be sure the child is cured", by four (19.0%). Twelve (51.1%) of these children were taken to HCF/chemist within 24 hours of onset of fever (4/6; intervention and 8/15; control arm) and nine (42.9%) others thereafter.

There was marked reduction in number of cases of malaria attended to during the intervention period compared to baseline based on the entry in the attendance registers in the health centres. However, record keeping by the health facilities was found to be grossly inadequate in this LGA.

## Discussion

The results of this study showed that provision of health education on malaria can improve caregiver's knowledge of malaria. The level of knowledge of causation, symptoms, and signs of malaria, treatment and prevention determines the extent to which appropriate steps are taken to prevent occurrence and treat a case adequately. During this study, knowledge of causation and prevention of malaria by caregivers were found to be poor at baseline. These findings are similar to those of past studies conducted in rural areas in Nigeria and other endemic countries where level of education of mothers was generally low [17,21,22]. This is in contrast to studies carried out in literate communities where as high as 82% of caregivers correctly related mosquito bite to malaria [23-26].

There was no concordance between method of prevention and perceived causes of malaria mentioned by the mothers. The expectation is that their prevention practices will be targeted towards perceived causes. This corroborates previous reports that peoples' thought about preventing malaria and preventing mosquito bites were not congruent [21,25]. The improvement in knowledge in the intervention arm of this study demonstrated the positive impact of health education on caregivers' knowledge and provided further evidence for the use of trained lay people to deliver educational messages to community members [9,14,16].

A high proportion of mothers in both arms of the study made presumptive diagnosis of malaria in their febrile children. This was more frequent in the intervention arm post intervention. When presumptive diagnosis and results of microscopy obtained during the follow-up study nested into this study were compared, the sensitivity and specificity of caregivers' diagnosis were 78.1% and 29.2% versus 82.3% and 8.3% in the intervention and control arms respectively (reported in another manuscript submitted for publication). This is encouraging because as many as eight out of ten cases of malaria were correctly diagnosed by mothers and if prompt and correct treatment is given the morbidity and mortality of children from malaria will be reduced drastically. This is in support of the WHO policy that all febrile illness in children < 5 years of age in malaria endemic areas be treated as malaria [2]. On the other hand however, the high frequency of presumptive diagnosis of malaria in febrile children could be dangerous for those children who did not have malaria as this would lead to delay in seeking appropriate treatment.

The finding in this study also demonstrated that it is possible to remarkably improve mothers'/caregivers' adherence to chloroquine treatment for children through provision of health education by trained lay people in the community and distribution of treatment guideline. The approach taken in this study was similar to that used for studies carried out to improve home treatment practices of caregivers in some other endemic countries in sub-Saharan Africa [13-17]. The improvement in level of adherence from 2.6% to 53.5% in the intervention arm of this study is well over the target of 50% increase and compared well with those reported in some past studies [9,13,14,17]-. In a controlled health facility based study by Okonkwo et al., (2001) adherence in the intervention group was 73.3% compared to 36.5 in the control group. Salako et al., (2001) reported compliance increased from 14.3% to 26.7%. In the study by Adeniyi et al., improved compliance from 27.8% to 98.1% was reported while Pagnoni et al reported an increase in compliance by number of doses of CQ administered from 0.8% to 12.9% and in terms of correct duration of treatment from 5.6% to 18.9%. In all the above studies compliance was measured by recall. In addition, Okonkwo et al., did volumetric measurement of the left over chloroquine syrup.

This study unlike those mentioned, provided a treatment guideline which included story presented as cartoon to help with early diagnosis and referral as well as dosing schedule for chloroquine use in uncomplicated malaria to all households. Also, special drug or pre-pack drugs were not provided to mothers; they were requested to buy antimalarial drugs from their usual drug source. Compliance was measured by recall as was the case in the earlier studies.

In this study, daily monitoring of patients for the nested follow-up study was carried out for nine months. Research assistants were withdrawn from the communities for three months prior to conduct of the post intervention survey. The frequent presence of research assistants in the study communities could have influenced the use of the guideline by caregivers and subsequently the correct use of chloroquine. The increase in proportion of those who used CQ correctly in the control arm could also be attributed to the frequent visit of research assistants. This is more so that no form of intervention or malaria control programme was carried out in the control health district. However, it was presumed that the three months absence of assistants from the field would be enough to reduce the influence of the team's presence on compliance. Other factors that may be responsible are the repeated health education provided by "mother trainers" to the intervention communities and the availability of the treatment guideline to every household to which mothers could refer.

The significant association of the use of the treatment guideline with correct use of chloroquine confirms the effectiveness of the guideline to improve compliance. This was also associated significantly with improved cure rate. This demonstrates that mothers could be trained to use chloroquine correctly at home by providing a simple guide. The fact that the use of the guideline was significantly associated with the level of education suggests that having some form of education provided a probable advantage in understanding of the guideline. However, this was not reflected in the correctness of the use of chloroquine as this was not significantly associated with the level of education of respondents. While it is possible that the positive impact of training a core group of mothers and producing guidelines on malaria treatment is maintained with the switch of first line drug to ACT, this may not be straight forward as the dose regimen for some ACTs is different from that of CQ. Hence, the applicability of the intervention to ACT use at community level needs to be tested.

Despite the fact that this study unlike others did not provide CQ to mothers/caregivers, the use of CQ increased in the intervention arm after intervention. This could be that more mothers opted to buy CQ subsequent to improved knowledge of treatment of malaria and the confidence in their ability to use CQ correctly [14]. The drug vendors who were also provided training and the treatment guideline could have increased the sale of antimalarial drug subsequent to the training they also had [15] and possibly increase demand by the caregivers.

The proportion of correct use of chloroquine was highest among those who treated malaria at home with drugs bought from drug sellers. It is also of note that most of them started treatment within 24 hours of noticing fever in the children. Mothers/caregivers who took ill children to chemist or health facility started later than 24 hours. This corroborates findings in past studies in southwest and southeast Nigeria [5,13] and confirms the feasibility of treating malaria promptly and correctly at home with adequate provision of effective health education and provision of guideline to mothers.

The relatively poor compliance by those who consulted at health facility and chemist could be a reflection of the inadequate knowledge of the attendants and consequently poor provision of information on drug use to mothers. This underscores the need to train drug distributors on the correct use of chloroquine and also to encourage positive change in their attitudes. Marsh et al. in an earlier study had reported that shopkeepers constitute an effective channel for intervention to the community. In the study by Marsh et al., plasma drug levels of children treated by shop keepers were assayed to determine the appropriateness and correctness of chloroquine sold and dispensed over-the-counter after training. The design of this study allowed mothers to continue with the usual practice of purchasing drugs from vendors trying to uphold the real context of home management of malaria (HMM). This however could be a draw back as the quality of drugs brought from the vendors may be sub-optimal.

One possible effect of sub-standard drug is that many of the prompt and seemingly correct treatments may not be beneficial to the health of the child and could also encourage development of resistance to drug by parasites. However, this was refuted by the cure rate observed in the follow-up study nested into this study. The overall D14 cure rate was 76.8% and by site, it was 91.3% in the intervention arm and 63.3% in the control site. The significant difference in cure rate in the two arms of the study demonstrates the positive effect of the intervention and the importance of compliance in achieving positive outcome with chloroquine treatment. It also support the use of over-the -counter bought chloroquine for malaria treatment. However, compliance by caregivers must be emphasized and ensured. Inclusion of drug stability test, measurement of drug plasma levels [15] and measurement of treatment outcome (such as cure rate) using drugs bought from vendors as done in this study will provide strong evidence for either continuing the use of drug vendors to distribute chloroquine for HMM or discouraging it. Other ways to encourage sale of quality drugs is through training and monitoring of the activities of the drug vendors.

## Conclusion

This study confirms earlier reports that majority of treatment for malaria take place in the home with drugs bought from drug vendors. It also provided evidence for the effectiveness of community-based approach to malaria treatment. "Mother trainers" were found to be effective in the delivery of health education messages and remarkable improvement in adherence to drug treatment of children suffering from malaria was achieved. The use of treatment guideline to complement training stands to further improve knowledge of mothers and adherence to malaria treatment with chloroquine at home. It is worthwhile to evaluate this mode of intervention (health education plus treatment guideline developed using participatory approach) in the use of artemisinin based combination drugs such as artemether-lumefantrine which is now the drug of choice in the treatment of acute uncomplicated malaria in Nigeria.

## Authors' contributions

IO and AMJ conceived of the study, IO carried out the field work and drafted the manuscript; OO contributed to the design of the study, supervised the field work and revised the manuscript draft considerably. CO supervised the laboratory work and revised the manuscript. EA supervised the analysis of the data and participated in the design of the study. All authors read and approved the final manuscript.

## Supplementary Material

Additional file 1 Assessment of a treatment guideline questionnaire – Appendix 1. This is the questionnaire used to collect data for this study. Click here for file
