# Supplementary material for: Assessment of a treatment guideline to improve home management of malaria in children in rural south-west Nigeria
Source: Malar J. 2008 Jan 29;7:24. doi: 10.1186/1475-2875-7-24 (PMC2268701; doi:10.1186/1475-2875-7-24)
Supplement: Additional file 1 — Assessment of a treatment guideline questionnaire – Appendix 1. This is the questionnaire used to collect data for this study. [file 1475-2875-7-24-S1.doc]

**DEVELOPMENT OF A TREATMENT GUIDELINE FOR HOME MANAGEMENT OF MALARIA IN CHILDREN**

**INSTRUMENT B STUDY NO________________**

ELIGIBLE MOTHERS’ QUESTIONNAIRE

TO BE ADMINISTERED TO ALL ELIGIBLES IN THE HOUSEHOLD

Greetings,

We are researchers from the University College Hospital, Ibadan. The purpose of this research is to determine mothers/caregivers’ knowledge about malaria and their treatment practice. The information to be gathered from this interview will guide us in the planning of a health education programme to improve caregivers’ knowledge and ability to treat a child with malaria correctly. Be confident that all the information you provide in this interview will be made confidential and used only for the purpose of this research.

| IDENTIFICATION | |
| --- | --- |
| HOUSEHOLD IDENTIFICATION NUMBER (WARD/HOUSE NO) |  |
| HOUSEHOLD QUESTIONNAIRE NUMBER (HIN/HHOLD ROSTER NO) |  |
| HOUSEHOLD ADDRESS |  |
| NAME OF RESPONDENT |  |

| INTERVIEWER VISITS | | | |
| --- | --- | --- | --- |
|  | 1 | 2 | FINAL VISIT |
| DATE  INTERVIEWER'S NAME  INTERVIEWER'S No  RESULT    How many eligible mothers? |  |  |  |
|  |  |  |  |
|  |  |  |  |
|  | * | * | * |
|  | * CODES  1. COMPLETE 4. REFUSED  2. NOT AT HOME 3. POSTPONED  5. OTHER--------- | | |

STUDY NO______________

**SECTION 1. BACKGROUND OF RESPONDENT**

Q101. Date of birth, if known? Day:____ Month______ Year _____

(probe: if day, month or year of birth are unknown, leave blank)

(if date is known, skip to Q103)

Q102. How old are you? ___________years 77. DK

Q103. Highest level of educationi?

1. None 2. Primary School 3. Secondary 4. Post secondary

Q104. Religion?

1.Christian 2. Muslim 3.Traditional religion 4. Omiran___________

Q105. Marital status ?

1. Single 2. Married 3. Co-habiting 4. Separated 5. Widow 6.Others _________

Q106. Occupation ?

1. House wife 2. Trader 3. Farmer 4. Junior civil servant 5. Senior civil servant 6. Teacher 7. Artisan/apprentice 8. Professional 9.Not employed 10. Others________

Q107. Who is the key person that takes care of children when they are sick?

1. Father 2. Mother 3. Maternal grand-parents 4. Paternal grand-parents

5. Both father and mother 88. Others ______________

Q108.Who takes the final decision on where to take a child to when he/she is sick?

1. Father 2. Mother 3. Maternal grand-parents 4. Paternal grand-parents

5. Both father and mother 88. Others ______________

**SECTION 2. KNOWLEDGE ON MALARIA AND ITS PREVENTION**

Q201. Fever is common among children; please tell us some of the illnesses that could cause fever in children.

_____________________________________ ______________________________________

Q202. What type of illness do you think your child could be having whenever he/she develops fever. 1.Malaria 2. Pneumonia 3. Measles 4. Typhoid

5. Schistosomiasis 6. Teething 88. Others ______________

Q203. Please tell us what you know about malaria by type (uncomplicated/severe), signs and symptoms, season it most occurring, how children get malaria and the treatment.

| Type | Sign Apere re **(fi ami* si eyi ti o je apere julo)** | Season it occurs most | How children contract malaria. | Treatment of malaria |
| --- | --- | --- | --- | --- |
|  | 1.  2.  3.  4.  5.  6.  77. DK |  | 1.  2.  3.  4.  5.  6.  77.DK |  |
|  | 1.  2.  3.  4.  5.  6.  77. DK |  | 1.  2.  3.  4.  5.  6.  77.DK |  |

Q204. Can malaria be prevented?

1.Yes 2. No 77. Don’t know [If yes go to Q 206)

Q205. If no, why did you say malaria cannot be prevented?

--------------------------------

Q206. If yes to Q 204, please answer the following question on methods of preventing malaria. (Interviewer, please do not probe)

| Ways to prevent malaria (circle)  (put asterisk* on the most important one) | Which ways do you use  (circle) | Is there any difficulty in using the method you use?(do not prompt) | |
| --- | --- | --- | --- |
| 1.Outpure of mosquto Apo efon  2. Do not allow stagnant water mase je ki o mi dagun  3. Drug to prevent malaria n ati dena iba  4. Do not allow children to stay long in the sunmase je ki omo pe ninu orun  5. fi ogun efon tabi tan ogun efon  6. fi ogun efon para  88. omiran-----------  77. nko mo/ nko le so  Combination ___________ | 1. Apo efon  2. mase je ki o mi dagun  3. Ogun ati dena iba  4. mase je ki omo pe ninu orun  5. fin ogun efon tabi tan ogun efon  6. fi ogun efon para  7. Nko lo nkankan  88. omiran-----------  77. nko mo/ nko le so  Combination_____________ | 1. cost  2. power failure  3. orun ogun efon  4. oru apo efon  5. nko ki nri ra  88. omiran _________  7. None  77.DK  Combination_______ | |
|  | |  |  |

**SECTION 3. FEBRILE EPISODE/MALARIA (CHILD THAT HAD FEBRILE ILLNESS LAST)**

(complete for each child < or 10yrs from household roster, starting with the youngest child)

Name of child _____________________ Household number_________________

Q301. Age: _____________________ SEX: (1) MALE (2) FEMALE

Q302. When did .........[name of child] had fever last? _________

Q302b. Last episode of fever occureed within the last two weeks? 1. Yes 2. No

Q303. When ...........had fever last, how long did it last?

1. Has fever at time of interview 2. 1-3 days 3.. 4-6 days

4. One to two weeks 77. Don’t know 88. Others______________

Q304. What are the other signs and symptoms (apart from the fever) ...........had?

1. Yes 2. No 77. Don’t know

a. chills/rigors 1 2 77

b. Diarrhoea 1 2 77

c. Vomiting 1 2 77

d. Cough 1 2 77

e. Difficultywith breathing 1 2 77

f. Convulsion 1 2 77

g. Skin rash 1 2 77

h. Restless 1 2 77

i. Weakness/not playing 1 2 77

j. Headache 1 2 77

k. Yellow eyes 1 2 77

l. Anorexia 1 2 77

88. Others ____________________________________________

Q305. What type of illness do you think ........had when he/she had fever last?

1. Malaria _____________________

2. Measles 3. Pneumonia 4. Yellow fever 5. Typhoid

6. Ordinary cough 77. Don’t know 88. Others_______

(If not malaria, GO TO Q307)

Q306. Do you think the malaria he had was severe?

1. Yes 2. No 77. Don’t know

Q307. What are the signs and symptoms of severe malaria that you know?

(circle all responses) (do not prompt)

1. Chills/rigors 2. Convulsion 3. Child not eating/anorexia 4. Persistent fever

5. Diarrhoea 6. Very high fever 7. Breathless 8. Yellow eyes

9. Palor 10. Coca cola urine 77. Don’t know 88. Others____________

Q308. What was the first treatment you gave to ............... ........

1. Gave herbs at home (Ask Q410- Q443)

2. Child taken to traditional medicine man (Ask Q 444 - Q453)

3. Gave orthodox drugs at home (Ask Q 455- Q485)

4. Took child to chemist / PMS (Aks Q486- Q4994)

5. Took child to the health centre in the distric /dispensary (Ask Q4995 - Q49910)

**INTERVIEWER PICK THE QUESTIONNAIRE ON TREATMENT OPTIONS (OPTIONS 1-5 questionnaires) AND ADMINISTER THE ONE TO THE OPTION MENTIONED. Thereafter, continue with the questions below.**

Q458. When you want to buy drug for achild with febrile illness, which of these is most important? (Only one response)

1. Paracetamol 2. Antimalarial (CQ, Fansidar etc) 3. Haematinics/vitamins

4. Butazolidine/phensic 5. Antibiotic

Q459. Why did you think the drug you mentioned is the most important in treating malaria? ___________________________

_____________________________

____________________________

STUDY NO______

**DEVELOPMENT OF A SIMPLE GUIDELINE FOR HOME MANAGEMENT OF MALARIA IN CHILDREN**

**TREATMENT OPTION 1: Caregiver treated child at home using traditional medicine**

Q410. How many hours after noticing fever did you commence the traditional medicine for .....? ___________

Q411. Where did you get the medicine from?

1. Prepared at own by yourself

2. Got it from a traditional healer (Go to Q 414)

Q412. How did you know how to prepare the medicine?

1. Inherited it from fore fathers

2. Taught by a traditional healer

3. Learned it from relations and/or friends

88. Others___________________

Q413. Name the herbs used to prepare the medicine.

1. __________________________________________

2. ____________________________________________

3. _________________________________________

Q414. How many times in a day did you adminster the medicine? ____________________

Q415. What is the measure of medicine given to a child under five years?

___________________teaspoon/day (quantify in mls)

Q416. What is the measure of medicine given to a child 5-10 years?

__________________teaspoon(mls) /day

Q417. For how many days did you give the medicine? _____________________

Q418. Did you at anytime had to use other drugs with the herbal medicine?

1. Yes 2. No 77. Don’t know

Q419. If yes to Q418, state the the other drugs used?

_______________________________________

Q420. Why did you have to use other drus in addition to the herval medicine?

_______________________________________

Q421. What was the outcome of the treatment you gave ........i?

1. Got well

2. Got a little better

3. There was no improvement in child’s condition Ko si iyato ninu aisan

4. Sickness got worse

Q422. Did you have to take .......... to a health facility, dispensary or chemist after treatment at home with herbal medicine?

1. Yes 2. No

Q423. If Yes to Q417, why?

1. Sickness persisted 2. To be sure child is cured

3. Sickness getting worse 4. Herbal medicine not effective

88. Others____________________

Q424. How many hours after noticing fever in ....... did you take him/her to health facility? ______________________hours

**DEVELOPMENT OF A SIMPLE GUIDELINE FOR HOME MANAGEMENT OF MALARIA IN CHILDREN**

STUDY NO______

**Treatment Option 2: Child taking to a traditional healer/herbalist**

Q425. How many hours after noticing fever did you commence the traditional medicine for .....? ___________

Q426. For how many days did you give the medicine? _____________________

.

Q427. If yes to Q418, state the the other drugs used?

_______________________________________

Q428. Why did you have to use other drus in addition to the herval medicine?

_______________________________________

Q429. What was the outcome of the treatment you gave ........?

1. Got well

2. Got a little better

3. There was no improvement in child’s condition Ko si iyato ninu aisan

4. Sickness got worse

Q430. Did you have to take .......... to a health facility, dispensary or chemist after treatment at home with herbal medicine?

1. Yes 2. No

Q431. Did you at anytime had to use other drugs with the herbal medicine?

1. 1. Yes 2. No 77. Don’t know

Q432. If Yes to Q417, why?

1. Sickness persisted 2. To be sure child is cured

3. Sickness getting worse 4. Herbal medicine not effective

88. Others____________________

Q433. How many hours after noticing fever in ....... did you take him/her to health facility? ______________________hours

**DEVELOPMENT OF A SIMPLE GUIDELINE FOR HOME MANAGEMENT OF MALARIA IN CHILDREN**

STUDY NO_______

**Treatment Option 3: Treated child at home using drugs bought from chemist, patent medicine sellers or drug hawkers.**

Q434. How many hours after noticing fever did you commence the traditional medicine for .....? ___________

Q435. What drug (s) did you buy to treat ....Nigbati e ra ogun lati se itoju ti o ni aisan iba, ewo ni e ra? (Do not prompt)

1. Nivaquine/Chloroquine |_| 2. Paracetamol/alabukun |_|

3. Aspirin |_| 4. Antibiotics (ogun kokoro) |_|

5. Phenergan/piriton |_| 6. Vitamin/blood tonic |_|

7. Assorted (“Akapo”) |_|

88. Others______________________ 77. Don’t know

Q436. Which of the following drug sellers did you buy drugs from?

1. Chemist/PMS 2. Drug hawkers 3. Dispensary / health centre

Q437. Fun awon ogun ti e lo fun omo yin yi, dahun awon ibeere yi

| Name of drug | Formulation  1. Syrup  2. Tablet  3. Injection | Dose | How many times a day | Duration | How may hours after noticing fever did you commence drug |
| --- | --- | --- | --- | --- | --- |
|  |  |  |  |  |  |
|  |  |  |  |  |  |
|  |  |  |  |  |  |

Q467**. FOR INVESTIGATOR - CORRECTNESS OF USE OF ANTIMALARIA. 1. YES 2. NO**

Q467b. Did you use the treatment guideline to treat ________ in this sickness? (Ask at post- intervention survey)

1. Yes 2. No

Q438. How satisfied were you with the drug(s) you brought to treat......?

1. Very satisfied 2. Satisfied 3. 50/50

4. Not satisfied 5. Not satisfied at all

Q439. Did you have to take .......... to a health facility, dispensary or chemist after treatment at home with orthodox drugs?

1. Yes 2. No

Q440. If Yes to Q417, why?

1. Sickness persisted 2. To be sure child is cured

3. Sickness getting worse 4. Herbal medicine not effective

88. Others____________________

Q441. How many hours after noticing fever in ....... did you take him/her to health facility? ______________________hours

**DEVELOPMENT OF A SIMPLE GUIDELINE FOR HOME MANAGEMENT OF MALARIA IN CHILDREN**

STUDY NO___________

**Treatment option 4: Took child to a chemist**

Q442. How many hours after noticing fever did you take child to a chemist.? ___________

Q443. How did the chemist attendant know the type of illness your child had? (Go to Q489)

1. Ask for what I noticed in child 2. Examined the child

3. Did a laboratory test (blood and urine test,e.t.c) 4. Checke the temperature of the child

88. Others __________________

Q444. What drugs did the attendant prescribed?

1.___________________

2. _____________________

3. _____________________

Q445. Did the attendant tell you how to use the drug(s)?

1. Yes 2. No

Q446. If no to Q.... , how did you know how the dose to give to your child?

1. Read the literature in the drug pack

2. Ask from a friend/neighbour

3. Used it as I did in the past

88. Others__________________________

Q447. How did you use the drug (s)?

| Name of drug | Formulation  1. Syrup  2. Tablet  3. Injection | Dose | How many time a day | Duration | Cost of drug | How many hours after noticing fever did you use drug |
| --- | --- | --- | --- | --- | --- | --- |
|  |  |  |  |  |  |  |
|  |  |  |  |  |  |  |
|  |  |  |  |  |  |  |

Q467.  **FOR INVESTIGATOR - CORRECTNESS OF USE OF ANTIMALARIA**

1. YES 2. NO

Q467b. Did you use the treatment guideline to treat ________ in this sickness? (Ask at post- intervention survey)

1. Yes 2. No

Q448. How satisfied were you with the drug(s) you brought to treat......?

1. Very satisfied 2. Satisfied 3. 50/50

4. Not satisfied 5. Not satisfied at all

Q449. Did you have to take .......... to a health facility, dispensary or chemist after treatment at home with orthodox drugs?

1. Yes 2. No

Q450. If Yes to Q417, why?

1. Sickness persisted 2. To be sure child is cured

3. Sickness getting worse 4. Herbal medicine not effective

88. Others____________________

Q451. How many hours after noticing fever in ....... did you take him/her to health facility? ______________________hours

**DEVELOPMENT OF A SIMPLE GUIDELINE FOR HOME MANAGEMENT OF MALARIA IN CHILDREN**

**STUDY NO________**

**Treatment option 5: Took child to health centre.**

Q452. How many hours after noticing fever did you take child to a chemist.? ___________

Q453. What drugs did the attendant prescribed?

1.___________________

2. _____________________

3. _____________________

Q454. Did the health worker tell you how to use the drug(s)?

1. Yes 2. No

Q455. If no to Q.... , how did you know how the dose to give to your child?

1. Read the literature in the drug pack

2. Ask from a friend/neighbour

3. Used it as I did in the past

88. Others__________________________

Q456. How did you use the drug (s)?

| Name of drug | Formulation  1. Syrup  2. Tablet  3. Injection | Dose | How many time a day | Duration | Cost of drug | How many hours after noticing fever did you use drug |
| --- | --- | --- | --- | --- | --- | --- |
|  |  |  |  |  |  |  |
|  |  |  |  |  |  |  |
|  |  |  |  |  |  |  |

Q467.  **FOR INVESTIGATOR - CORRECTNESS OF USE OF ANTIMALARIA**

1. YES 2. NO

Q467b. Did you use the treatment guideline to treat ________ in this sickness? (Ask at post- intervention survey)

1. Yes 2. No

Q457. How satisfied were you with the drug(s) you treatment received at the health centre......?

1. Very satisfied 2. Satisfied 3. 50/50

4. Not satisfied 5. Not satisfied at all
